# The efficacy of lyticase and β-glucosidase enzymes on biofilm degradation of *Pseudomonas aeruginosa* strains with different gene profiles

**DOI:** 10.1186/s12866-019-1662-9

**Published:** 2019-12-12

**Authors:** Maryam Banar, Mohammad Emaneini, Reza Beigverdi, Rima Fanaei Pirlar, Narges Node Farahani, Willem B. van Leeuwen, Fereshteh Jabalameli

**Affiliations:** 1 Department of Microbiology, School of Medicine, Tehran University of Medical Sciences, Tehran, Iran; 2 Leiden Centre for Applied Bioscience, University of Applied Sciences Leiden, 2333CR Leiden, Netherlands

**Keywords:** *Pseudomonas aeruginosa*, β-Glucosidase, Lyticase, Biofilm

## Abstract

**Background:**

*Pseudomonas aeruginosa* is a nosocomial pathogen that causes severe infections in immunocompromised patients. Biofilm plays a significant role in the resistance of this bacterium and complicates the treatment of its infections. In this study, the effect of lyticase and β-glucosidase enzymes on the degradation of biofilms of *P. aeruginosa* strains isolated from cystic fibrosis and burn wound infections were assessed. Moreover, the decrease of ceftazidime minimum biofilm eliminating concentrations (MBEC) after enzymatic treatment was evaluated.

**Results:**

This study demonstrated the effectiveness of both enzymes in degrading the biofilms of *P. aeruginosa*. In contrast to the lyticase enzyme, β-glucosidase reduced the ceftazidime MBECs significantly (*P* < 0.05). Both enzymes had no cytotoxic effect on the A-549 human lung carcinoma epithelial cell lines and A-431 human epidermoid carcinoma cell lines.

**Conclusion:**

Considering the characteristics of the β-glucosidase enzyme, which includes the notable degradation of *P. aeruginosa* biofilms and a significant decrease in the ceftazidime MBECs and non-toxicity for eukaryotic cells, this enzyme can be a promising therapeutic candidate for degradation of biofilms in burn wound patients, but further studies are needed.

## Background

*Pseudomonas aeruginosa* is a nosocomial pathogen that causes severe infections in patients with cancer, cystic fibrosis (CF), and burn injuries [1, 2]. It accounts for about 10% of nosocomial infections [3]. Biofilm production of *P. aeruginosa* is an essential factor in the persistence of infections [4]. The biofilm matrix of *P. aeruginosa* is composed of various exopolysaccharides (alginate, Psl, and Pel), extracellular DNA, and proteins [5, 6]. Alginate is a polymer containing mannuronic acid and guluronic acid [7], which is produced by the mucoid phenotype of *P. aeruginosa* in the lungs of CF patients and associated with chronic infections and antibiotic resistance [4, 8]. Several different genes are involved in encoding exopolysaccharides alginate, Psl, and Pel. Twelve structural genes located on the *alg*D gene operon are involved in the biosynthesis of alginate. The *alg*D gene encodes a GDP- mannose dehydrogenase enzyme converting GDP-mannose to the GDP-mannuronic acid that controls the alginate production [6]. The Psl polysaccharide constitutes a repeating pentamer of D-mannose, L-rhamnose, and D-glucose monosaccharides [9]. Studies revealed that Psl is involved in cell-cell and cell-surface interactions of bacteria in biofilm and makes the scaffold for biofilm formation. It also mediates the resistance of biofilm to phagocytosis and the host immune clearance [9, 10]. The Psl is encoded by a 15 co-transcribed gene cluster (*psl*A to *psl*O), that 11 out of 15 genes are necessary for Psl production [11]. The *psl*B gene encodes Phosphomannose Isomerase/ GDP-mannose pyrophosphorylase (PMI-GMP), which is a bifunctional enzyme and contributes to the synthesis of Psl precursors. Studies have shown that *psl*B mutants can still produce the Psl polysaccharide. The *psl*D gene encodes for a periplasmic protein that mediates the transmission of Psl polymer across the periplasmic space. Mutations in this gene result in a defect in Psl production [9]. Pel is a glucose-rich polysaccharide that plays a role in biofilm formation, maintenance, and resistance of biofilm against antibiotics, especially aminoglycosides [12].. The Pel operon (*pel*A-G) encodes seven proteins involved in the production of Pel polysaccharide, which all of them are important in Pel biosynthesis. The *pel*F gene encodes a cytoplasmic protein that is a glycosyltransferase and involves the transmission of sugar units of Pel as well as the Pel polymerization [13].

Because biofilm in *P. aeruginosa* is a relevant factor that confers resistance to environmental stresses, phagocytic defenses, antimicrobial agents, and xenobiotics [14–17], and due to the increasing intrinsic resistance of this bacterium to many antibiotics, treatment of its infections are limited [13]. So researchers are trying to find new therapeutic approaches against infections of this bacterium [18]. In recent years, the anti-biofilm ability of enzymes against biofilms of *P. aeruginosa* was studied [6, 17]. For example, deoxyribonuclease (DNase) enzyme that has a specific effect on degrading the DNA in the mucus or sputum of CF patients [19, 20] is used as a therapy for CF patients with the commercial name of Pulmozyme [21]. Alginate lyase is another enzyme that can degrade the biofilm of *P. aeruginosa* [22] and is a promising candidate for the treatment of infections caused by mucoid strains of this bacterium [23].

The ability of lyticase and β-glucosidase enzymes to degrade β (1 → 3) and β (1 → 4) bonds between the glucose units [24, 25] and their probable effects on Psl and Pel polysaccharides were the reasons for choosing these enzymes.

This study aimed to investigate the destructive effect of lyticase and β-glucosidase enzymes on biofilms of clinical strains of *P. aeruginosa*.

## Results

Among the 122 *P. aeruginosa* clinical strains isolated from independent patients with CF or burn wound infections, a total of 11 strains were selected based on their unique gene profiles (presence or absence of the biofilm exopolysaccharides encoding genes: *psl*B, *psl*D, *alg*D, and *pel*F) and phenotypic traits (the state of biofilm formation and susceptibility to amikacin and ceftazidime) (Table 1). It was tried to select strains from all gene profiles (there were eight different gene profiles) with the ability to generate strong or moderate biofilms and susceptibility to amikacin and ceftazidime. However, since there were few strains in some groups, these criteria were not applicable in some cases. For example, although strains BR3, 4, 5, and 6 were resistant to amikacin and ceftazidime, because of their unique gene profiles were enrolled in the enzymatic analysis.

**Table 1:** Gene profiles and phenotypic characteristics of *P. aeruginosa* strains that were evaluated in this study (*n* = 11)

Strain	Gene profile	Resistance pattern	Biofilm production
BR1^a^	*pelF*^*+*^*, algD*^*+*^*, pslB*^*+*^*, pslD*^*+*^	–	strong
BR2	*pelF*^*+*^*, algD*^*+*^*, pslB*^*+*^*, pslD*^*+*^	–	strong
CF1^b^	*pelF*^*+*^*, algD*^*+*^*, pslB*^*+*^*, pslD*^*+*^	–	strong
BR3	*pelF*^*−*^*, algD*^*−*^*, pslB*^*−*^*, pslD*^*−*^	AK, CAZ	weak
BR4	*pelF*^*−*^*, algD*^*−*^*, pslB*^*−*^*, pslD*^*−*^	AK, CAZ	strong
BR5	*pelF*^*−*^*, algD*^*−*^*, pslB*^*−*^*, pslD*^*−*^	AK, CAZ	strong
BR6	*pelF*^*−*^*, algD*^*+*^*, pslB*^*+*^*, pslD*^*+*^	AK	strong
CF	*pelF*^*+*^*, algD*^*−*^*, pslB*^*+*^*, pslD*^*+*^	–	strong
CF3	*pelF*^*+*^*, algD*^*+*^*, pslB*^*−*^*,*	–	strong
BR7	*pelF*^*+*^*, algD*^*+*^*, pslB*^*−*^*, pslD*^*−*^	C	strong
BR8	*pelF*^*−*^*, algD*^*+*^*, pslB*^*−*^*, pslD*^*−*^	AK, CAZ	moderate

*AK* Amikacin, *CAZ* Ceftazidime

^a^BR; Burn strain

^b^CF; Cystic fibrosis strain

**Fig. 1:**
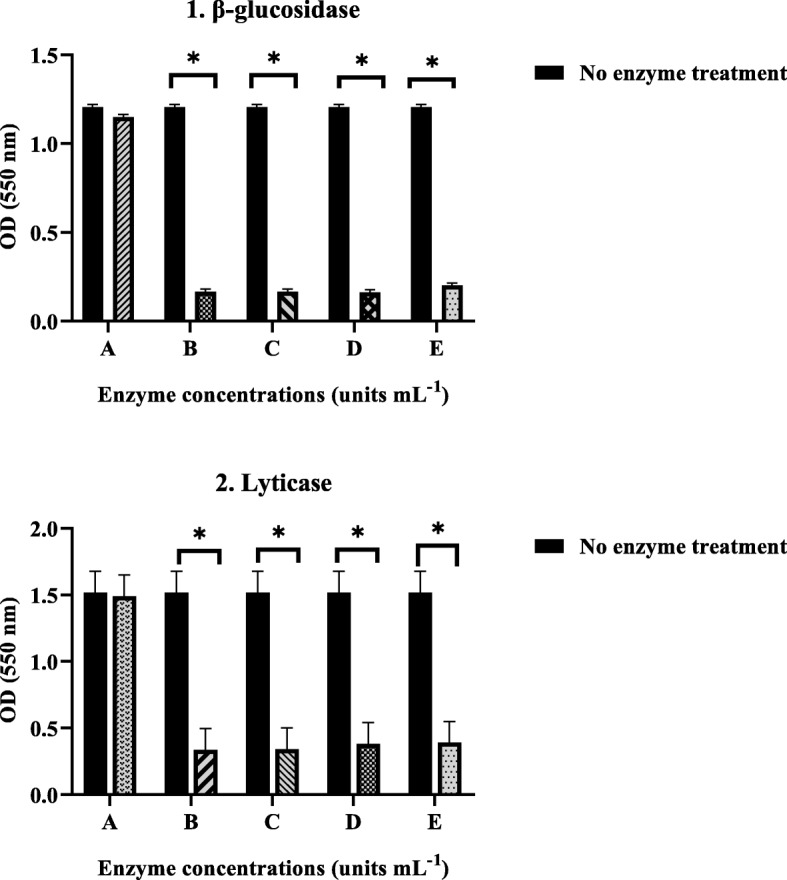
The effects of serial dilutions of (1) enzyme β-glucosidase [0.025 (**a**), 0.05 (**b**), 0.1 (**c**), 0.2 (**d**), and 0.4 (**e**) units mL^− 1^] and (2) enzyme lyticase [1.25 (**a**), 2.5 (**b**), 5 (**c**), 10 (**d**), and 25 (**e**) units mL^− 1^] on biofilms of *P. aeruginosa* strain BR1 that were evaluated by CV assay. The experiment was done once in triplicates. Error bars represent standard deviation (SD). Asterisks indicate the statistically significant difference with control (no enzyme treatment) (*P < 0.05)

**Fig. 2:**
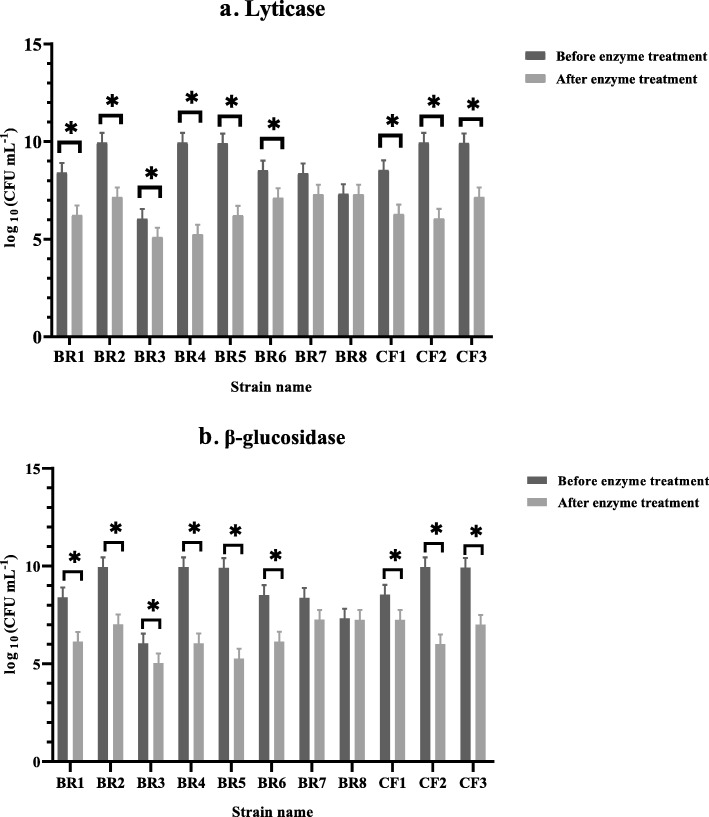
The effects of selected concentrations of enzymes (**a**) lyticase (2.5 units mL^− 1^) and (**b**) β-glucosidase (0.05 units mL^− 1^) against biofilm embedded *P. aeruginosa* strains with different gene profiles that were determined by colony counting technique. Error bars represent standard deviation (SD). Asterisks indicate the statistically significant difference before and after enzyme treatment (*P < 0.05)

**Fig. 3:**
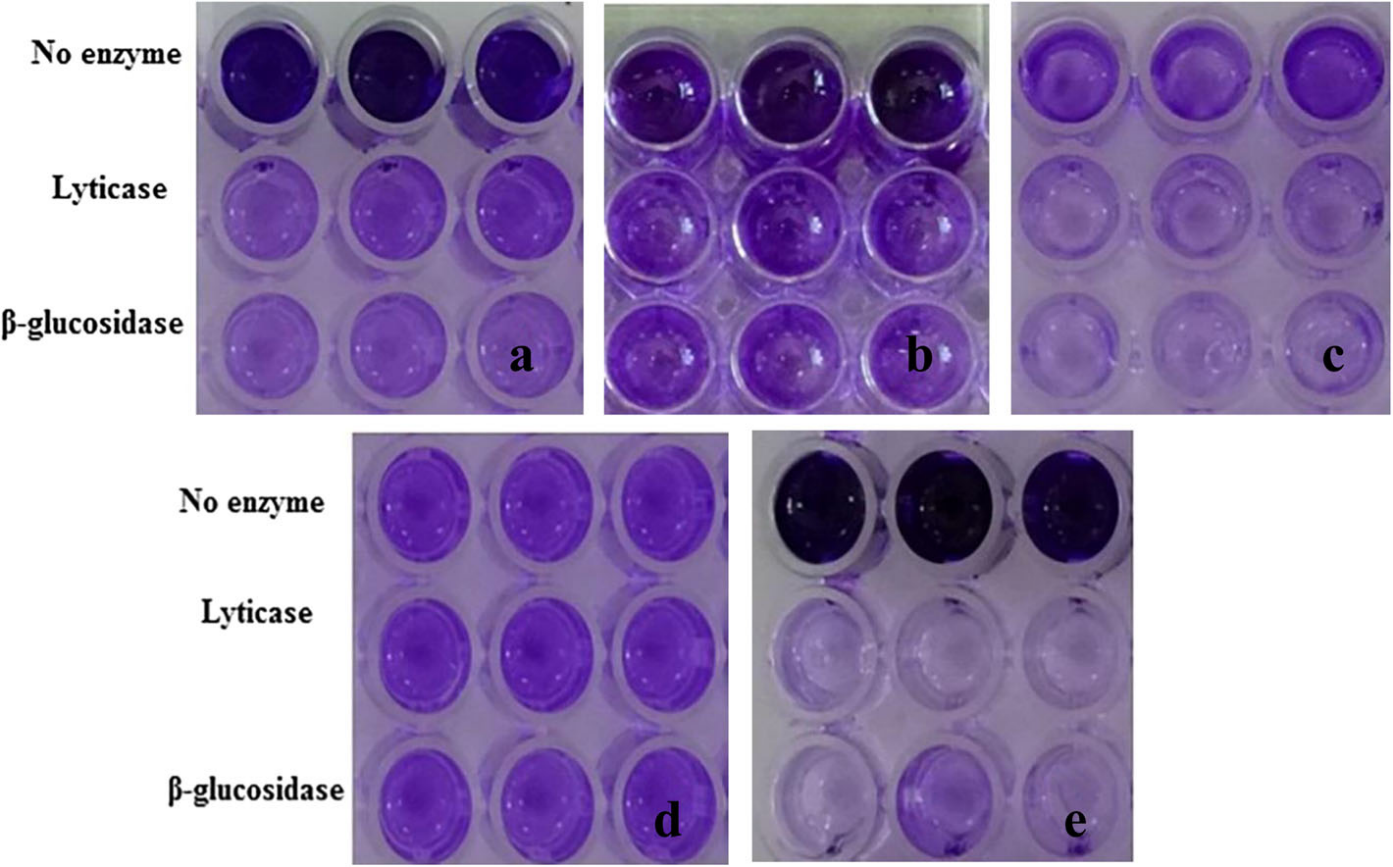
The effects of selected concentrations of enzymes lyticase (2.5 units mL^− 1^) and β-glucosidase (0.05 units mL^− 1^) against biofilm embedded *P. aeruginosa* strains with different gene profiles: **a** strain BR1, **b** strain BR7, **c** strain BR3, **d** strain BR8, and **e** strain CF3

For example, the degradative effect of serial dilutions of lyticase and β-glucosidase enzymes on the biofilm of strain BR1 is shown in Fig. 1. Both enzymes had a destructive effect on biofilms and reduced OD (optical density) values (*P* < 0.05). The most effective concentrations of lyticase and β-glucosidase enzymes on the biofilm were 2.5 units mL^− 1^ and 0.05 units mL^− 1^, respectively (Fig. 1). The effect of the most effective concentrations of enzymes on the biofilms of *P. aeruginosa* strains with different gene profiles was evaluated using crystal violet and colony counting methods (Table 2, Fig. 2, and Fig. 3). As shown, there was an agreement between the results of the two assays, and the two examined enzymes destroyed the biofilm and resulted in the reduction of the colony-forming units (CFU) (Data are available in Additional file 1).

**Table 2:** Effect of selected concentrations of enzymes lyticase (2.5 units mL^-1^) and β-glucosidase (0.05 units mL^-1^) on the biofilm of *P. aeruginosa* strains with different gene profiles that were assessed by CV assay

	Lyticase (2.5 units mL^-1^)		β-glucosidase (0.05 units mL^-1^)	
Strain	Before enzyme treatment	After enzyme treatment	Before enzyme treatment	After enzyme treatment
BR1^a^	Strong	Weak	Strong	Weak
BR2	Strong	Moderate	Strong	Weak
BR3	Weak	Negative	Weak	Negative
BR4	Strong	Negative	Strong	Weak
BR5	Strong	Negative	Strong	Negative
BR6	Strong	Moderate	Strong	Weak
BR7	Strong	Moderate	Strong	Moderate
BR8	Moderate	Moderate	Moderate	Moderate
CF1^b^	Strong	Weak	Strong	Moderate
CF2	Strong	Weak	Strong	Negative
CF3	Strong	Moderate	Strong	Weak

^a^*BR* Burn strain

^b^*CF* Cystic fibrosis strain

**Fig. 4:**
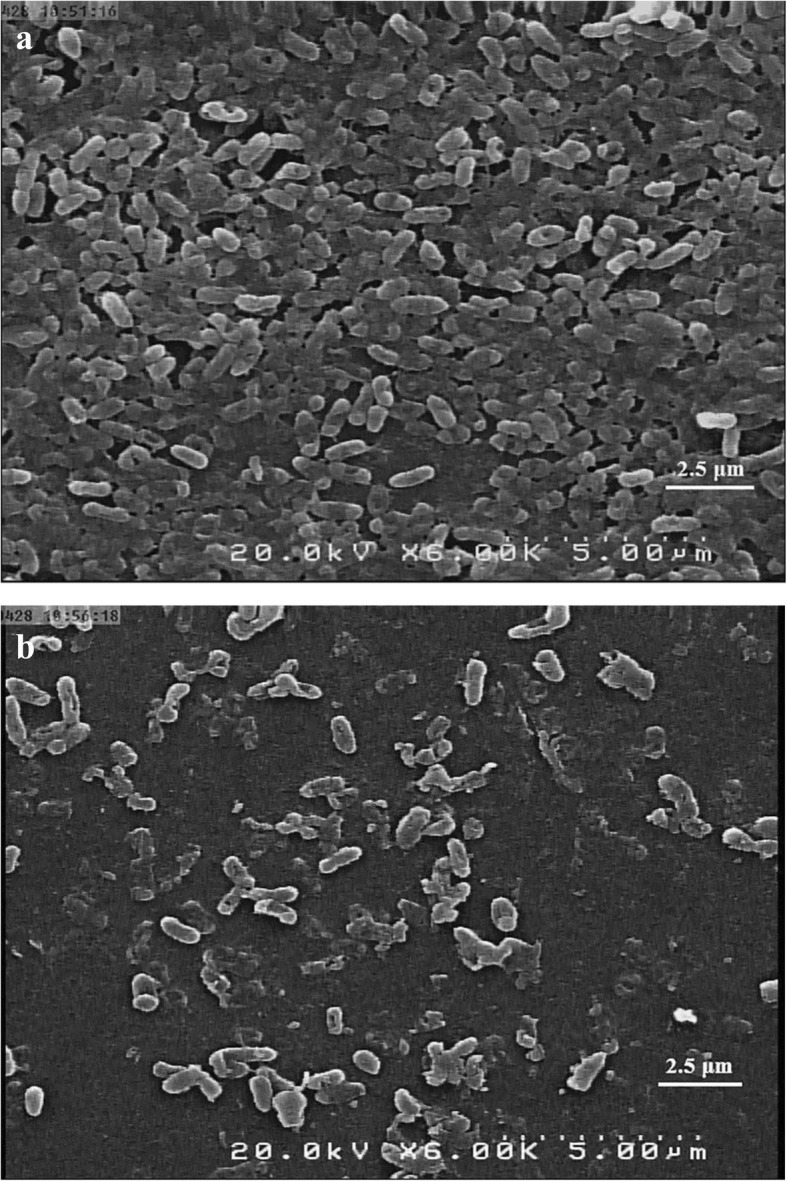
The scanning electron microscopy (SEM) images of *P. aeruginosa* biofilms (strain BR3). **a** Untreated control biofilm. **b** After 1 h treatment with enzyme β-glucosidase (0.05 units mL^− 1^) (6000x magnification)

After testing the effect of selective concentrations of enzymes on *P. aeruginosa* biofilms, the SEM analysis (scanning electron microscopy) was applied to visualize the biofilm degrading effect of the selected concentration of β-glucosidase enzyme (0.05 units mL^− 1^) (Fig. 4).

The bactericidal effects of enzymes on *P. aeruginosa* planktonic cells were investigated. Accordingly, the β-glucosidase was toxic and killed bacterial cells so that no turbidity was seen in the wells, and no colonies were present (MIC and MBC for all strains < 0.025 units mL^− 1^). But the lyticase enzyme had no bactericidal effect, and bacterial cells grew after treatment with this enzyme (MIC and MBC for all strains > 50 units mL^− 1^).

Amongst 11 strains, six susceptible strains to ceftazidime and amikacin in their planktonic states (BR1, 2, 7, and CF1, 2, 3) were selected to estimate the minimum inhibitory concentration (MIC) and the minimum biofilm eliminating concentrations (MBEC) of these antibiotics. Strain BR6, which was resistant to amikacin, and strains BR3, 4, 5 and 8, which were resistant to both amikacin and ceftazidime, were not involved in the experiment (Table 3). Results demonstrated that biofilm formation led to increasing ceftazidime resistance significantly (*P* < 0.05); however, strains in the biofilm state remained susceptible to the amikacin.

**Table 3:** Comparison of the results of Minimum Inhibitory Concentration (MIC) and Minimum Biofilm Eliminating Concentrations (MBECs) for *P. aeruginosa* strains

Strain	Amikacin (μg mL^−1^)		Ceftazidime (μg mL^−1^)	
	MIC	MBEC	MIC	MBEC
BR1	4	16	2	1024
BR2	4	16	2	1024
BR7	8	8	2	1024
CF1	8	8	1	512
CF2	2	8	2	512
CF3	8	16	4	1024

^a^*BR* Burn strain

^b^*CF* Cystic fibrosis strain

The effects of the simultaneous use of lyticase or β-glucosidase with ceftazidime on the MBEC values of ceftazidime are shown in Table 4. Results showed that lyticase had no significant effect on the MBEC values of ceftazidime (*P* > 0.05). However, β-glucosidase significantly reduced the ceftazidime MBECs (*P* < 0.05).

**Table 4:** The combined effects of enzymes and ceftazidime on the MBEC values of ceftazidime

Strain	Ceftazidime (CAZ) (μg mL^−1^)	CAZ+ lyticase (μg mL^− 1^)	CAZ + β-glucosidase (μg mL^− 1^)
BR1	1024	1024	512
BR2	1024	512	8
BR7	1024	512	32
CF1	512	512	128
CF2	512	512	16
CF3	1024	512	8

**Fig. 5:**
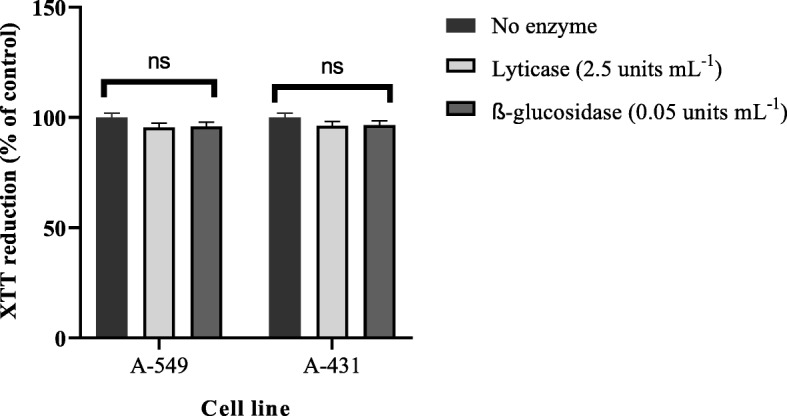
Influence of the most effective concentration of enzymes lyticase (2.5 units mL^− 1^) and β-glucosidase (0.05 units mL^− 1^) on the viability of the A-549 human lung carcinoma epithelial cell lines and A-431 human epidermoid carcinoma cell lines after 24 h incubation. The experiment was done two times in duplicates. Error bars represent standard deviation (SD). The ns represent non-significant difference compared with control (no enzyme treatment) (*P* > 0.05)

The cytotoxicity assay revealed that lyticase and β-glucosidase had no significant effect on mitochondrial activity and cell viability (*P* > 0.05) (Fig. 5) (Data are available in Additional file 1).

## Discussion

In recent years, enzymes have been considered as potential anti-biofilm and debriding agents [26, 27]. There are several examples of enzymes used as anti-biofilm agents in treating the infections or wound debridement, including debridase (bromelain- derived), collagenase (clostridio peptidase A), trypsin, streptokinase, lysozyme, fibrinolysin and dispersin B [28–33]. These enzymes can be used in debridement of wounds and removing barriers that debilitate wound healing, such as devitalized tissues, scars, and bacterial biofilms [30–33].

In this study, the influence of lyticase and β-glucosidase enzymes on biofilms of different *P. aeruginosa* strains was investigated. The results demonstrated that both lyticase and β-glucosidase enzymes degraded the biofilms of *P. aeruginosa* strains with various gene profiles, altered the state of biofilms, and decreased the CFUs significantly. However, they did not destroy the biofilms of the two strains BR7 and BR8 with gene profiles of *pelF*^*+*^*, algD*^*+*^*, pslB*^*−*^*, pslD*^*−*^ and *pelF*^*−*^*, algD*^*+*^*, pslB*^*−*^*, pslD*^*−*^, respectively (*P* > 0.05). Regarding the gene profiles of strain BR7, it seems that this strain lacks Psl polysaccharide in its biofilm structure, which is one of the substrates of lyticase and β-glucosidase enzymes. Neither of the tested enzymes destroyed the biofilm of strain BR8 since these enzymes did not have any effect on the structure of alginate.

The β-glucosidase enzyme was highly efficient on ceftazidime MBECs and reduced the MBEC 2 to 128 times. But, the lyticase enzyme was not efficient and reduced the ceftazidime MBECs only by a factor two. Probably, the lyticase degradation fragments had an inhibitory effect and prevented the ceftazidime activity on the bacterial cells in the remaining biofilm. For that reason, the ceftazidime MBECs showed no significant changes. On the other hand, β-glucosidase had a bactericidal effect, in contrast to lyticase. Maybe this is another reason that the lyticase did not reduce the antibiotic resistance of bacterial biofilms.

According to the toxicity assay, both enzymes were non-toxic and did not affect the mitochondrial cell functions. Future studies concerning the assessment of the toxicity of these enzymes on primary cell cultures and animal models are needed.

There are some limitations to this study. First, the total sample size was small (122 strains) and when strains classified based on their gene profiles, the number of strains in some groups was very few. This point can affect statistical analysis and conclusion. Second, the expression of biofilm encoding genes (*pslB*, *pslD*, *algD*, and *pelF*) and the structure of biofilm in selected strains was not evaluated. Therefore, the effect of enzymes on the biofilm of strains with different gene profiles was discussed based on the assumptions.

## Conclusion

In conclusion, considering the features of the β-glucosidase enzyme, including notable degradation of *P. aeruginosa* biofilms and a significant decrease in the ceftazidime MBECs and non-toxicity for eukaryotic cells, this enzyme can be a promising candidate as an anti-biofilm agent. So it is recommended to perform further studies on it. Given the polymicrobial nature of biofilms, it is suggested to investigate the efficacy of the β-glucosidase enzyme on the degradation of mixed-species biofilms. Moreover, the effect of lyticase enzyme on the MBECs of other antibiotics can be evaluated in the subsequent studies. Also, in future studies, the effects of these enzymes on the development of immune responses in the body and on the skin and respiratory tract normal flora, should be examined.

## Methods

### Bacterial strains

A total number of 122 *P. aeruginosa* clinical strains which were isolated from independent patients with CF or burn wound infections were collected from clinical microbiology laboratory of a tertiary hospital in Tehran, Iran. Strains were identified as *P. aeruginosa* using standard biochemical tests, including Gram stain, oxidase, catalase, oxidation-fermentation (OF), and the Kligler Iron Agar (KIA) tests [34]. Eleven out of 122 strains based on their gene profiles (presence or absence of the biofilm exopolysaccharides encoding genes: *pslB*, *pslD*, *algD*, and *pelF*) and phenotypic traits (the state of biofilm formation and susceptibility to amikacin and ceftazidime) were selected and included in the study.

### Antimicrobial susceptibility testing

Amikacin and ceftazidime are the antibiotics of choice that are used for the treatment of CF or burn patients with *P. aeruginosa* infection. The susceptibility of *P. aeruginosa* to both antibiotics was assessed using the disk diffusion agar method according to the Clinical and Laboratory Standards Institute (CLSI) guidelines [35]. *Escherichia coli* ATCC 25922 was used as the quality control of the test.

### Molecular detection of genes encoding biofilm exopolysaccharides

The presence of genes encoding biofilm exopolysaccharides in *P. aeruginosa* (*algD, pelF, pslB, and pslD*) was established by the PCR (polymerase chain reaction) assay, as previously described [6]. The specific oligonucleotide primer F-5′—GCG.AGT.TTC.TCC.TCA.ACA.CC-3′ and R-5′-CGA.CCG.TAG.ATG.TCG.TTG.AA-3′ was used for detection of the *pslB* gene.

### Biofilm analysis

The biofilm-forming capacity of the strains was determined in triplicate using a colorimetric microtiter plate assay (CV assay) as described previously [6]. *Pseudomonas aeruginosa* ATCC 27853 and sterile broth were used as the positive and negative controls, respectively. The bacterial cells were inoculated with turbidity equal to 0.5McFarland standard (1.5 × 10^8^ CFU mL^− 1^). The ODs of the wells was measured at 550 nm using a microplate reader (Anthos Labtec instruments, type: 22550). All the assays were done in triplicate and repeated three times for each strain.

Three standard deviations above the mean absorbance of negative control were considered as cut-off OD (OD_C_). Biofilm formation was classified into four different groups using the following formulas: If OD < ODc, the biofilm was not formed (negative), If ODc < OD < 2xODc, the biofilm was weak, If 2xODc < OD < 4xODc, the biofilm was moderate. If 4xODc < OD, the biofilm was strong.

### Determination of the enzyme activity

In this study, the degradable effect of the two enzymes β-glucosidase and lyticase was evaluated. The β-glucosidase enzyme is purified from almonds and is involved in the hydrolysis of β-glycosidic bonds connecting carbohydrate residues in β-D-glycosides. It converts cellobiose and cello-oligosaccharides to glucose [24].

The lyticase enzyme is purified from gram-positive bacteria, *Arthrobacter luteus*, which is a member of the Micrococcaceae family. It hydrolyzes poly-β (1 → 3)-glucose bonds such as yeast cell wall glucan and generates spheroplasts from fungi for transformation. It also is used for DNA extraction from yeast cells [25].

The biofilm detachment potency of the enzymes was determined by Kaplan et al., [36]. In short, the wells were rinsed once with sterile normal saline, and different concentrations of lyticase enzyme [1.25, 2.5, 5, 10 and 25 units mL^− 1^] and β-glucosidase enzyme [0.025, 0.05, 0.1, 0.2 and 0.4 units mL^− 1^] were added to the wells [24]. After incubation for 1 h at 37 ° C, wells contents were removed and were washed three times with normal saline and stained with crystal violet (CV assay) [6]. In our study, biofilms without any enzymatic treatment and medium with neither bacteria nor enzyme were considered as test controls. The assay was performed once in triplicate for each enzyme concentration. Further studies were performed by the most effective concentrations of each enzyme. Enzymes were purchased from Sigma Aldrich (St Louis, USA).

The destructive effect of selected concentrations of each enzyme on the biofilms of *P. aeruginosa* strains was also determined by colony counting technique [37]. For each strain and enzyme, the assay was done once in triplicate. The effect of the selected concentration of β-glucosidase (0.05 units mL^− 1^) on the biofilm of *P. aeruginosa* was observed by a scanning electron microscope (SEM) as described by Nemoto K et al.*,* [19].

The bactericidal effect of the enzymes on planktonic cells of *P. aeruginosa* was evaluated as described before [6]. Briefly, a serial dilutions of enzymes lyticase [1.25, 2.5, 5, 10, 25, and 50 units mL^− 1^] and β-glucosidase [0.025, 0.05, 0.1, 0.2, 0.4, and 0.8 units mL^− 1^] were added to the wells containing 100 μL of Mueller—Hinton broth and bacterial suspension (final inoculum density of 10^8^ CFU mL^− 1^). The selected concentrations of the enzymes were in the range of biofilm degradation concentrations. After the incubation, the effect of enzymes on the bacterial growth checked, and the MIC and MBC (minimum bactericidal concentration) values were determined. The MBC value of an enzyme was considered as the lowest concentration of the enzyme that killed 99.9% of the bacterial cells [38]. Each experiment was carried out three times for all strains.

### Determination of minimum inhibitory concentration (MIC) and minimum biofilm eliminating concentration (MBEC)

The MIC of amikacin (Sigma Aldrich, St Louis, USA) and ceftazidime (Jaber Ebne Hayyan Co, Iran) for the six sensitive selected strains was evaluated by broth microdilution method (antibiotic concentrations ranging from, 0.5 through 256 μg mL^− 1^) according to the CLSI guidelines [35]. Biofilms were established, and then each well was washed three times with normal saline to remove unbound bacteria to determine the MBECs of amikacin and ceftazidime for biofilm cultures. Subsequently, 100 μL of a given antibiotic concentration in cation-adjusted Muller–Hinton Broth was added to each well [39]. The plate was incubated at 37 ° C for 20 h, followed by adding 50 μL of fresh XTT labeling mixture [2,3-bis[2-methyloxy-4-nitro-5-sulfophenyl]-2H-tetrazolium-5-carboxanilide (XTT)] (Roche, Germany) to each well and incubated for 1 h at 37 ° C under dark condition [40]. The MBEC value was determined as the lowest concentration of the antibiotic that inhibited the re-growth of bacteria from the treated biofilm [41].

### The effect of the combination of enzymes and ceftazidime on *P. aeruginosa* biofilms

The combined effect of enzymes and ceftazidime on *P. aeruginosa* biofilms was determined as previously described [42, 43]. The MBEC values of ceftazidime for biofilm cultures were determined by the XTT reduction assay.

### Cytotoxicity assay

#### Cell line and culture

The cytotoxic effect of enzymes was investigated in A-549 human lung carcinoma epithelial cell lines (IBRC C10080) and A-431 human epidermoid carcinoma cell lines (NCBI Code: C204).

#### Cell viability assay

Mitochondrial functions of the cells after 24 h exposure to the enzymes were evaluated by the XTT reduction assay. After 24 h exposure to the enzymes, specific amounts of XTT labeling mixture were directly added to the culture wells. After 4 h incubation in the dark conditions, the absorbance at 492 nm was measured with a microplate reader. In this study, control positive wells were cells with no enzyme exposure, and control negative wells contained a sterile medium. Each experiment was performed in duplicate.

### Statistical analysis

Distributions of variables (ODs of biofilms) were evaluated using the Kolmogorov-Smirnov test. Since variables showed a non-normal distribution, a Wilcoxon Signed Ranks test was applied for comparison of ODs before and after treatment with enzymes and a Kruskal-Wallis test was used for determining the effects of enzymes on different *P. aeruginosa* strains. The differences between ceftazidime MBECs before and after using enzymes were evaluated using the Mann-Whitney U test for each strain. A *P*-value < 0.05 was considered statistically significant. All tests were performed using an online free available Graph Pad software (http://www.graphpad.com).

## Supplementary information

**Additional file 1.** Supplementary data.

## Data Availability

All data generated or analyzed during this study are included in this published article [and its Additional files].

## Abbreviations

CF: Cystic fibrosis
CFU: Colony forming units
CLSI: Clinical and Laboratory Standards Institute
CV assay: Crystal violet assay
KIA: Kligler Iron Agar
MBEC: Minimum biofilm eliminating concentrations
MIC: Minimum inhibitory concentration
OD: Optical density
OF: Oxidation-fermentation
PCR: Polymerase chain reaction
SEM: Scanning electron microscopy
